# The role of context in implementation research for non-communicable diseases: Answering the ‘how-to’ dilemma

**DOI:** 10.1371/journal.pone.0214454

**Published:** 2019-04-08

**Authors:** Meena Daivadanam, Maia Ingram, Kristi Sidney Annerstedt, Gary Parker, Kirsty Bobrow, Lisa Dolovich, Gillian Gould, Michaela Riddell, Rajesh Vedanthan, Jacqui Webster, Pilvikki Absetz, Helle Mölsted Alvesson, Odysseas Androutsos, Niels Chavannes, Briana Cortez, Praveen Devarasetty, Edward Fottrell, Francisco Gonzalez-Salazar, Jane Goudge, Omarys Herasme, Hannah Jennings, Deksha Kapoor, Jemima Kamano, Marise J. Kasteleyn, Christina Kyriakos, Yannis Manios, Kishor Mogulluru, Mayowa Owolabi, Maria Lazo-Porras, Wnurinham Silva, Amanda Thrift, Ezinne Uvere, Ruth Webster, Rianne van der Kleij, Josefien van Olmen, Constantine Vardavas, Puhong Zhang, on behalf of the GACD Concepts and Contexts working group

**Affiliations:** 1 Department of Food, Nutrition and Dietetics, Uppsala University, Uppsala, Sweden; 2 Department of Public Health Sciences, Karolinska Institutet, Stockholm, Sweden; 3 Department of Community, Environment and Policy, University of Arizona, Tucson, Arizona, United States of America; 4 Global Alliance for Chronic Diseases, University College London, London, United Kingdom; 5 Department of Medicine, University of Cape Town, Rondebosch, South Africa; 6 Department of Family Medicine, McMaster University, Hamilton, Ontario, Canada; 7 School of Medicine and Public Health, The University of Newcastle, Callaghan, New South Wales, Australia; 8 Department of Epidemiology and Preventive Medicine, Monash University, Clayton, Victoria, Australia; 9 Department of Population Health, New York University School of Medicine, New York City, New York, United States of America; 10 The George Institute for Global Health, The University of New South Wales, Australia, Newtown New South Wales, Australia; 11 Collaborative Care Systems Finland, Helsinki, Finland; 12 University of Eastern Finland, Helsinki, Finland; 13 Department of Nutrition and Dietetics, Harokopio University, School of Health Sciences & Education, Kallithea, Athens, Greece; 14 Department of Public Health and Primary Care, Leiden University Medical Center (LUMC), Leiden, Netherlands; 15 Zena and Michael A. Wiener Cardiovascular Institute, Icahn School of Medicine at Mount Sinai, New York City, New York, United States of America; 16 Population Health Division, The George Institute for Global Health- India, New Delhi, Delhi, India; 17 Department of Basic Sciences, Universidad de Monterrey, Monterrey, Nuevo Leon, Mexico; 18 Centro de Investigacion Biomedica, Instituto Mexicano del Seguro Social, Monterrey, Nuevo Leon, Mexico; 19 Faculty of Health Sciences, University of the Witwatersrand, Centre for Health Policy, School of Public Health, Braamfontein, Johannesburg, South Africa; 20 Department of Endocrinology and Metabolism, All India Institute of Medical Sciences (AIIMS), Ansari Nagar, New Delhi, Delhi, India; 21 School of Medicine, College of Health Sciences, Moi University, Eldoret, Kenya; 22 European Network for Smoking and Tobacco Prevention, Brussels, Belgium; 23 Department of Medicine, University of Ibadan, Department of Medicine, University College Hospital, Ibadan, Nigeria; 24 Unidad de Conocimiento y Evidencia (CONEVID), CRONICAS Center of Excellence in Chronic Disease, Universidad Peruana Cayetano Heredia, Miraflores, Lima, Peru; 25 School of Public Health, Imperial College London, London, United Kingdom; 26 Department of Public Health, Institute of Tropical Medicine Antwerp, Antwerpen, Belgium; 27 University of Antwerp, Antwerpen, Belgium; 28 The George Institute for Global Health at Peking University Health Science Center, Beijing, China; University of Queensland, AUSTRALIA

## Abstract

**Introduction:**

Understanding context and how this can be systematically assessed and incorporated is crucial to successful implementation. We describe how context has been assessed (including exploration or evaluation) in Global Alliance for Chronic Diseases (GACD) implementation research projects focused on improving health in people with or at risk of chronic disease and how contextual lessons were incorporated into the intervention or the implementation process.

**Methods:**

Using a web-based semi-structured questionnaire, we conducted a cross-sectional survey to collect quantitative and qualitative data across GACD projects (n = 20) focusing on hypertension, diabetes and lung diseases. The use of context-specific data from project planning to evaluation was analyzed using mixed methods and a multi-layered context framework across five levels; 1) individual and family, 2) community, 3) healthcare setting, 4) local or district level, and 5) state or national level.

**Results:**

Project teams used both qualitative and mixed methods to assess multiple levels of context (avg. = 4). Methodological approaches to assess context were identified as formal and informal assessments, engagement of stakeholders, use of locally adapted resources and materials, and use of diverse data sources. Contextual lessons were incorporated directly into the intervention by informing or adapting the intervention, improving intervention participation or improving communication with participants/stakeholders. Provision of services, equipment or information, continuous engagement with stakeholders, feedback for personnel to address gaps, and promoting institutionalization were themes identified to describe how contextual lessons are incorporated into the implementation process.

**Conclusions:**

Context is regarded as critical and influenced the design and implementation of the GACD funded chronic disease interventions. There are different approaches to assess and incorporate context as demonstrated by this study and further research is required to systematically evaluate contextual approaches in terms of how they contribute to effectiveness or implementation outcomes.

## Introduction

Implementation science advances ‘what works’ to ‘what works where and why’, and specifically deals with “how to move evidence-based interventions (EBIs) into healthcare policy and practice” [1]. Context, in relation to implementing EBIs, is the environment or setting in which the proposed change is to be implemented [2]. Understanding context is crucial for successful implementation. However, EBIs are implemented in complex, multi-faceted and dynamic environments, which arguably means that the same intervention would rarely work in the same way in different contexts.

Fortunately, there are several existing frameworks [3–5] and tools [6,7] to help facilitate the structured and comprehensive conceptualization and assessment of context within the implementation of complex interventions. The Promoting Action on Research Implementation in Health Services (PARiHS) framework, for example, was developed to advance understanding of implementation as a multi-faceted process [2]. This three-dimensional framework emphasizes the relationship between: (a) the type of the evidence being used, (b) the ability of the context to cope with change and (c) the facilitation needed for a successful change process [5]. So while the tools and strategies used to implement an intervention are important, the context of implementation equally matters.

Moreover, Sheikh et al. outline the importance of going beyond measuring the concrete and tangible ‘hardware’ of the health system to capture the ‘software’, i.e. the contextual setting that drives the ideas, interests, values, norms and power relations underpinning health system performance [8]. The relevance of context in implementation and the need for contextualization is well-acknowledged, but the ‘how’ is not often clear. How we should explore or measure the salient features of context, let alone report and act on it, remains rather ambiguous. Luoto et al. found that in previous studies the reporting of context was, at best, ‘mostly fair or poor and highly variable’ among global health interventions [9]. The lack of context and implementation information is a major gap in the evidence needed by global health policymakers in their decision-making to assess whether or not an intervention applies to their setting.

Due to the increasing awareness of the complexity of implementation research, it is important to determine how context can be systematically explored, evaluated or incorporated in research projects [10,11]. Through this paper, we investigated how context was assessed in a group of implementation projects focusing on non-communicable diseases (hypertension, diabetes and lung diseases). Given the dearth of information on how to conduct research on context, this is not a best practice guide but a clear illustration of how investigators have explored, evaluated or incorporated context within their studies. Specifically, we have aimed to:

1. Describe the methods and the levels from individual to national/state at which context has been assessed (including exploration or evaluation) in Global Alliance for Chronic Diseases (GACD) funded implementation research projects focused on improving health in people with chronic diseases
2. Describe how contextual lessons were incorporated into the intervention or the implementation process.

## Methods

### Study setting: Global Alliance for Chronic Diseases

The Global Alliance for Chronic Diseases (GACD) was founded in 2009 and is a collection of the world’s largest public research funding agencies [12]. Currently the alliance includes 14 national or regional funding agencies across the globe. The goal of the GACD is to address the high burden of chronic diseases in low and middle-income countries (LMICs) and amongst vulnerable and indigenous populations in high-income countries (HICs) by facilitating implementation research through targeted research calls coordinated across all participating funding agencies. We focus on three of the programs from these calls: I. Hypertension Research Program (2012–17); II. Diabetes Research Program (2014–19); and III. Lung Diseases Research Program (2015–21). The GACD Research Network provides a forum through which early, mid and senior career level researchers funded through these programs can explore cross cutting themes related to implementation science. Several researchers active in the GACD Research Network formed a cross cutting theme group to explore the general issue of context across projects (Context and Concepts working group).

### Study design

**Fig 1:**
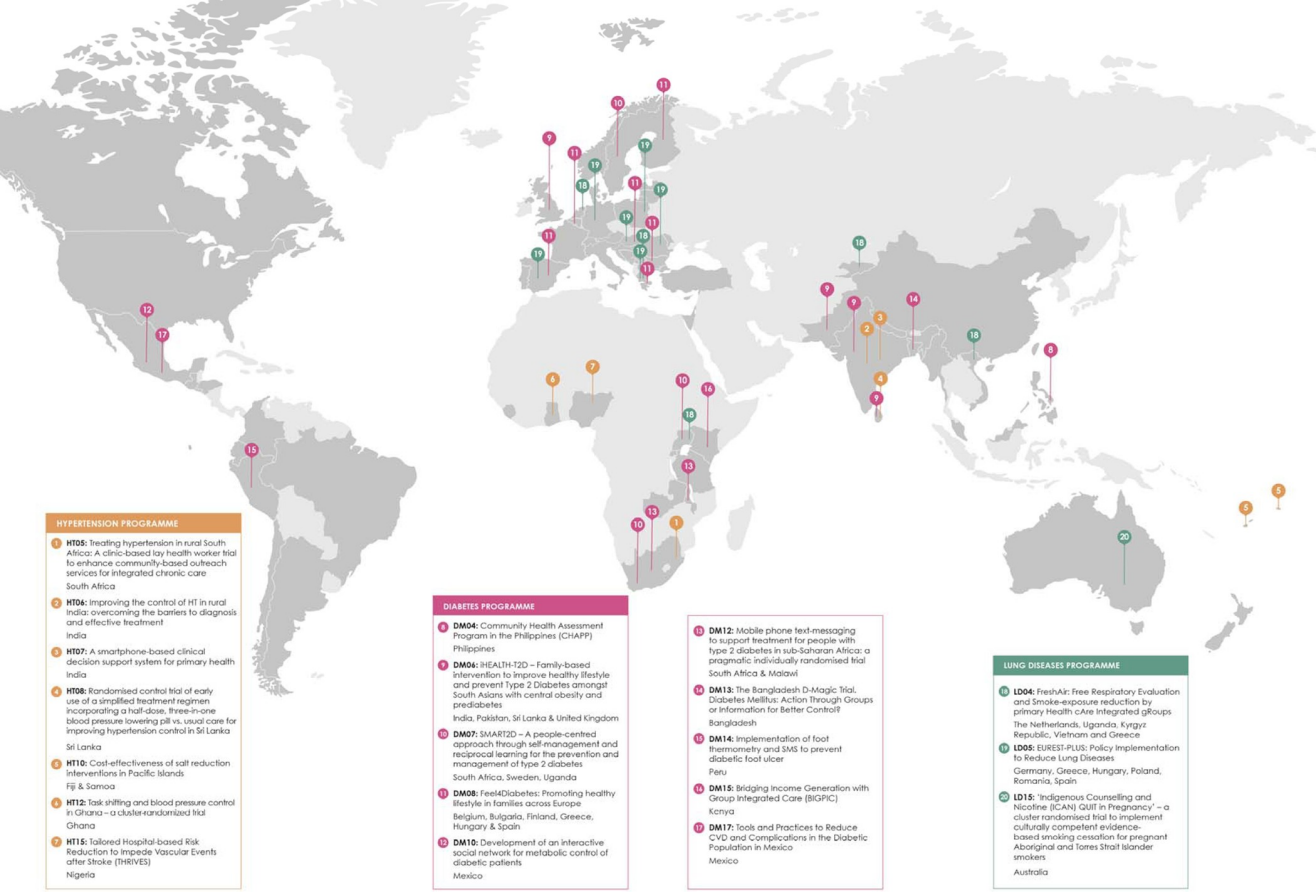
A map of the projects included in the study (n = 20).

This was a cross-sectional study with a semi-structured survey conducted across projects belonging to research programs I to III. Researchers from all projects in research programs I and II were invited to participate if their project included an intervention or implementation component and had reached the implementation stage (n = 28). Due to the timing of funding, research program III projects were invited if they included an intervention or implementation component and had already completed the intervention (development or testing) or implementation stage at the time of the survey (n = 3). Fig 1 illustrates the global locations of the studies. When specific projects are referred to in the text, we have used the official GACD codes, (e.g. HT05 (hypertension project #5), DM04 (diabetes project #4), LD04 (lung diseases project #4)) which can also be used to access specific project related information from www.gacd.org.

### Conceptual framework

#### Conceptual framework defining the levels and components of context

**Fig 2:**
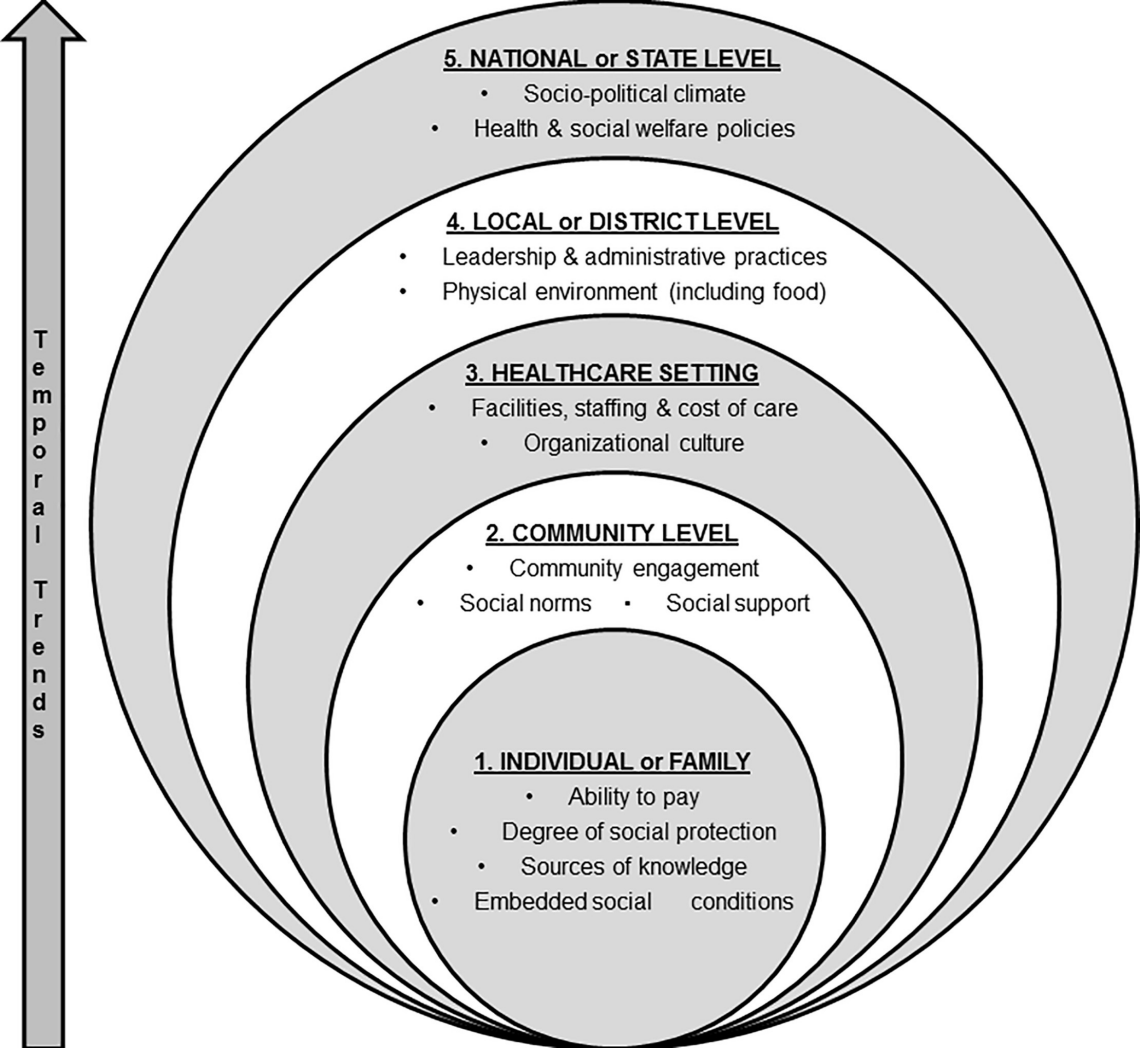
Multi-layered context framework.

The working group used a multi-layered context framework (Fig 2) inspired by Taplin et al. [13] developed for implementation research involving cancer. The inclusion of dimensions from the COACH tool by Bergström et al. [7] and work from Edwards and Barker [14] make the framework more relevant for chronic disease research across different settings. The framework reflects the complex nature of context and includes five different levels; 1) individual and family, 2) community, 3) healthcare setting, 4) local or district level, and 5) state or national level. Each level was further divided into sublevels that included ethical, legal, social and economic issues, as well as all stakeholders in the implementation environment (i.e. patients, policy makers, payers, and healthcare providers) [15]. Temporal trends were an overarching theme as it is applicable to any level of context. The working group agreed upon contextual sublevel components and their definitions prior to using them in the survey (see S1 Table for definitions).

### Data collection

#### Data collection tool

A semi-structured survey was developed to assess if and how projects were exploring or evaluating context; i.e., at which specific level, what methodologies were being used and how the data was being integrated back into the research project. It was structured by our adapted multi-layered context framework (Fig 2) and included both closed and open-ended questions at each level to quantify but also explore more in-depth how teams assessed and responded to context. The survey also quantified the frequency of different inter-linkages between the contexts and sub-components. The tool was discussed in the working group and agreement was reached on the type and scope of questions before it was piloted.

#### Data collection process

The working group piloted the survey in two waves with different projects participating in each round. Changes to the survey following the piloting rounds included: revisions to make completion of the survey less onerous and more intuitive, refining of the definitions around context, addition, separation and/or conflation of some factors within the various levels of context, and inclusion of instructions on how the various levels of context should be interpreted within the questionnaire. The groups that participated in the pilot were provided the opportunity to update their initial responses using the final version of the survey.

The principal investigator of each project identified one or two team members who had a comprehensive understanding of the project and worked directly with the development of the intervention and/or implementation. The survey was sent via email to identified team members and three reminders were deployed to ensure a high completion rate. The tool was administered in English which was the common language among the participating researchers. Data collection commenced February 2017 and ended July 2017.

### Data analysis

As a mixed-methods study, our survey used an embedded design [16] and included both quantitative (close ended) and qualitative (open ended) questions that were designed to complement each other in the analysis phase [17]. The quantitative data identified where efforts to assess context across studies were concentrated and the qualitative data identified assessment methods and ways in which findings were being incorporated into the study. The qualitative and quantitative data from the semi-structured survey were analyzed separately before being connected in the final stage and were displayed side-by-side, which is one of the documented modes of displaying mixed methods results [18]. *The quantitative survey data* were analyzed using descriptive statistics (frequencies and proportions) across the context sub levels. The Fisher’s exact test and t-tests were used to compare differences in proportions and means, respectively.

*The qualitative data* were extracted by one co-author (GP), then compiled and structured based on context (sub) levels by the first author (MD). Content analysis was used to guide data analysis [19,20]. The initial tasks of coding, grouping and condensing the text were undertaken independently by two members of the team (MI and MD). They reviewed the data and the preliminary results in person at annual meetings, in conference calls, and through email discussions. The preliminary results were presented at the sixth Annual Scientific Meeting (ASM) of the GACD in October 2017 to all ASM participants. This provided an opportunity to engage with other working group members and obtain feedback and suggestions. A major concern raised at the ASM pertained to the ability to verify the actions reported by the teams. It was therefore decided to use the ASM handbook’s annual progress reports submitted independently by each team to cross-reference and triangulate discrepancies [21]. MD and KSA then refined the codes further by reading the entire dataset multiple times to ensure that the data were coded consistently and subsequently condensing codes into broader categories and overarching themes. The themes were finalized between MI, MD and KSA. Reflexivity was accounted for throughout the analysis process by discussion between the main coders, examining one’s own biases, and by presenting the results to the larger group.

Any clarifications needed from specific teams were sought through direct questions to the individual teams during the manuscript review process. All comments were discussed with the main analysis team and any discrepancies were further discussed and resolved via email. General consensus on the results and major messages were obtained from the working group by email.

### Ethical considerations

The study presents aggregate data that was limited to describing research methods from an array of funded research projects. We do not have any human subjects’ data in the study or analyses, and thus we did not seek ethics review. All participating projects however, received ethical clearance from their respective institutions and other local authorities (e.g. Ministries/Municipalities) to conduct their own studies.

## Results

### Project participants’ characteristics

Thirty-one out of 49 projects from programs I, II and III met the eligibility criteria and 20 agreed to participate (response rate 65%): seven from hypertension, ten from diabetes and three from lung diseases (Table 1). Due to the nature and timing of the different funding calls, the projects were in different stages ranging from development of the intervention to implementation or evaluation. The contextual levels assessed by each project are outlined in Table 1.

**Table 1:** GACD study projects description and context level assessed.

GACD Code and Project Name	Research aim and levels of context assessed	Study Location	Study design to evaluate intervention/implementation	Target Population	Duration (yrs)	Funding Agency	Website
**HT05: Treating hypertension in rural South Africa: A clinic-based lay health worker trial to enhance community-based outreach services for integrated chronic care**	*Aim*: To reduce population levels of uncontrolled hypertension, especially in those individuals at greatest risk, by supporting and strengthening the management of hypertension in primary care clinics *Levels of context assessed*: Healthcare setting and local or district level	South Africa	Cluster randomized control trial using two population surveys to measure the primary outcome	Hypertension patients attending clinics included in the trial	3	MRC-UK	https://www.gacd.org/research-projects/hypertension/ht05
**HT06: Improving the control of HT in rural India: overcoming the barriers to diagnosis and effective treatment**	*Aim*: 1) To quantify and identify the determinants of the prevalence, awareness, treatment, and control of hypertension in three different rural populations in India, each at differing levels of the epidemiological transition. 2) Identify barriers to control of hypertension. 3) Develop and pilot intervention strategies to improve the control of hypertension. The pilot program was based on those factors identified as contributing to control of hypertension in these settings and includes both management and prevention strategies aimed at the individual, health service delivery and policy levels. *Levels of context assessed*: All five levels	India	Mixed methods approach comprising qualitative (interview, focus group discussion, intervention meeting reports) and quantitative data (survey, participant evaluation, post intervention outcome data) to determine feasibility of the proposed intervention model. There was also a census of health services.	Health care workers, research officers, participants with hypertension, and health services.	3	NHMRC	https://www.gacd.org/research-projects/hypertension/ht06
**HT07: A smartphone-based clinical decision support system for primary health**	*Aim*: 1) To develop a multifaceted primary healthcare worker intervention that utilizes a mobile device-based clinical decision support system to improve optimal BP control in high risk individuals. 2) To evaluate this program utilizing a mixed methods evaluation in a cluster randomized trial involving 54 villages in rural Andhra Pradesh. *Levels of context assessed*: All five levels	India	Mixed methods approach using a stepped- wedge cluster randomized, controlled trial (cRCT) to evaluate the effectiveness of the intervention	Non-physician health workers, doctors and participants with risk factors for cardiovascular disease	3	NHMRC	https://www.georgeinstitute.org/projects/systematic-medical-appraisal-referral-and-treatment-smart-health
**HT08: Randomized control trial of early use of a simplified treatment regimen incorporating a half -dose, three-in-one blood pressure lowering pill vs. usual care for improving hypertension control in Sri Lanka**	*Aim*: To investigate effectiveness, cost-effectiveness, and acceptability of Triple pill (Triple BP lowering therapy) compared to usual care for early management of high BP in Sri Lanka. *Levels of context assessed*: Individual or family and healthcare setting	Sri Lanka	Mixed methods approach using quantitative data for main trial outcomes, qualitative process evaluation (interviews with patients and health care providers) and cost effectiveness evaluation.	Adults with persistent hypertension requiring initiation or up-titration of blood pressure lowering therapy.	3	NHMRC	https://www.gacd.org/research-projects/hypertension/ht08
**HT10: Cost effectiveness of salt reduction interventions in Pacific Islands**	*Aim*: To evaluate the impact and cost-effectiveness of multi-faceted intervention strategies to reduce salt in the Pacific Islands. Specifically, to measure current salt consumption patterns, develop an intervention program to reduce salt in each country and then monitor progress against key indicators. *Levels of context assessed*: Individual or family, community, local or district level and state or national level	Fiji, Samoa	Mixed methods approach using sub-analysis of quantitative data for main trial outcomes, routine monitoring data, qualitative process evaluation stakeholder interviews) and cost effectiveness evaluation.	National populations in both Fiji and Samoa	4	NHMRC	https://www.gacd.org/research-projects/hypertension/ht10
**HT12: Task shifting and blood pressure control in Ghana—a cluster-randomized trial**	*Aim*: To evaluate the effectiveness of the implementation of the WHO Package (i.e. task-shifting strategy for hypertension (TASSH)) targeted at CVD risk assessment versus provision of health insurance coverage alone on BP reduction *Levels of context assessed*: Individual or family, community, healthcare setting, and local or district level	Ashanti Region, Ghana	Cluster randomized trial design at the health facility level	Patients with uncomplicated hypertension	5	NHLBI, NIH	https://www.gacd.org/research-projects/hypertension/ht12
**HT15: Tailored Hospital-based Risk Reduction to Impede Vascular Events after Stroke (THRIVES)**	*Aim*: To determine whether a culturally-sensitive multipronged post-discharge intervention can significantly reduce BP, enhance achievement of guideline recommended targets for risk factor control, and lower recurrent vascular events in Nigeria. *Levels of context assessed*: All five levels	Nigeria	Mixed methods approach that includes qualitative (key informant interviews, focus group discussion) and quantitative data (survey, participant evaluation, post intervention outcome data)	Clinicians, study participants and other intervention implementation team	5	NIH, NINDS	https://www.gacd.org/research-projects/hypertension/ht15
**DM04: Community Health Assessment Program in the Philippines (CHAP-P)**	*Aim*: To adapt the elements of the expanded CHAP-P intervention model to low—and middle-income countries (LMICs) and to determine the effect of the CHAP-P on the HbA1c levels of community residents in the Philippines. *Levels of context assessed*: All five levels	Philippines	Mixed methods approach using an RCT for main trial outcomes and qualitative and quantitative data gathered to better understand processes, outputs and outcomes	People at risk for diabetes (adults 40 years of age and over)	5	CIHR, IDRC	https://www.gacd.org/research-projects/diabetes/dm04
**DM06: iHEALTH-T2D - Family-based intervention to improve healthy lifestyle and prevent Type 2 Diabetes amongst South Asians with central obesity and prediabetes**	*Aim*: To compares lifestyle modification vs usual care for prevention of T2DM amongst non-diabetic South Asians with central obesity and / or prediabetes. *Levels of context assessed*: Individual or family, community, healthcare setting, and local or district level	India, Pakistan, Sri Lanka, United Kingdom	Cluster randomized trial	Non-diabetic South Asians (aged 40–70) with central obesity and / or prediabetes	5	EC	http://ihealth-t2d.eu/our-study-2/ihealth-t2d-study/
**DM07: SMART2D - A people-centred approach through Self-Management and Reciprocal learning for the prevention and management of Type-2-Diabetes**	*Aim*: To strengthen capacity for T2DM care (both prevention and management), through proven strategies like task-shifting to non-physician health care providers and community health workers and expanding care networks through community-based peer support groups. *Levels of context assessed*: Individual or family, community, healthcare setting, and local or district level	Uganda, South Africa, Sweden	Cluster randomized adaptive implementation trial. Mixed methods used: Quantitative data collection mainly at two-time points (0 & 12 months) and outcome, process and costing analysis; Qualitative data collection and analysis for formative research and process evaluation.	Adults with T2DM and pre-diabetes in low-resourced setting in Uganda (rural area) and South Africa (urban slums); Adults with or at high risk for T2DM in socio-economically disadvantaged suburbs in Sweden.	4	EC	http://ki.se/en/phs/smart2d
**DM08: Feel4Diabetes: Developing and implementing a community-based intervention to create a more supportive social and physical environment for lifestyle changes to prevent diabetes in vulnerable families across Europe**	*Aim*: To develop, implement and evaluate an evidence-based and potentially cost-effective and scalable intervention program to prevent T2DM among families from vulnerable groups across Europe. *Levels of context assessed*: All five levels	Belgium, Bulgaria, Finland, Greece, Hungary, Spain	Cluster randomized intervention. Quantitative data were collected at 3-time points (baseline, follow-up 1 and follow-up 2) to assess the impact and outcome of the intervention, during and after the intervention to assess its process and cost-effectiveness.	Vulnerable Families in six European countries.	4.5	EC	www.feel4diabetes-study.eu
**DM10: Development of an interactive social network for metabolic control of patients with diabetes**	*Aim*: To develop a smartphone application in order to minimize risk-related attitudes and in order to change the behavior towards their disease among people who suffer from T2DM. *Levels of context assessed*: Individual or family, community, healthcare setting, and local or district level	Mexico	Phenomenological qualitative research	Patients, practitioners, administrative staff	2	CONACYT	https://www.gacd.org/research-projects/diabetes/dm10
**DM12: SMS to support treatment for people with T2DM in sub-Saharan Africa: a pragmatic individually randomized trial**	*Aim*: To test the effectiveness of sending brief adherence support messages to patients (delivered by SMS text) in improving health outcomes and medication adherence in patients with T2DM. *Levels of context assessed*: All five levels	South Africa and Malawi	Mixed methods approach using quantitative data for main trial outcomes (RCT), qualitative process evaluation (interviews with patients and health care providers) and cost effectiveness evaluation.	Adults with T2DM	3.5	MRC-SA, MRC-UK	https://www.gacd.org/research-projects/diabetes/dm12
**DM13: The Bangladesh D-Magic Trial. Diabetes Mellitus: Action Through Groups or Information for Better Control?**	*Aim*: To evaluate the impact of a) a participatory community mobilization intervention and b) an mHealth health promotion and awareness intervention on the prevalence of intermediate hyperglycemia and diabetes and the two-year cumulative incidence of diabetes mellitus among individuals with intermediate hyperglycemia in rural Bangladesh. *Levels of context assessed*: All five levels	Bangladesh	Three arm cluster randomized controlled trial, cost-effectiveness survey and continuous mixed-methods process evaluation.	Adults aged 30 years and above in rural Faridpur district, Bangladesh.	3	MRC-UK	https://www.gacd.org/research-projects/diabetes/dm13
**DM14: Implementation of foot thermometry and SMS to prevent diabetic foot ulcer**	*Aim*: To compare the incidence of diabetic foot ulcer between the arm that receives thermometry alone and the arm that receives thermometry + messages (SMS and voice message). *Levels of context assessed*: Individual or family	Peru	Evaluator-blinded, randomized trial.	Individuals with T2DM, 18–80 years, having a present dorsalis pedis pulse in both feet, risk group 2 or 3 using the diabetic foot risk classification system	2	FIC, NIH	https://www.gacd.org/research-projects/diabetes/dm14
**DM15: Bridging Income Generation with Group Integrated Care (BIGPIC)**	*Aim*: To utilize a transdisciplinary implementation research approach to address the challenge of reducing CVD risk in low-resource setting by evaluating the effectiveness of group medical visits and microfinance groups for CVD risk reduction among individuals with diabetes or at increased risk for diabetes. *Levels of context assessed*: All five levels	Kenya	Mixed method approach with qualitative methods to assess contextual factors and four-arm cluster randomized trial to test the effectiveness of the intervention and cost-effectiveness analysis	Individuals with diabetes or at increased risk for diabetes in western Kenya	5	NHLBI, NIH	https://www.gacd.org/research-projects/diabetes/dm15
**DM17: Tools and Practices to Reduce CVD and Complications in the Diabetic Population in Mexico**	*Aim*: To assess the effectiveness of an adapted evidence-based community health worker intervention (Meta Salud Diabetes) aimed at reducing behavioral and clinical risk for CVD among adults with diabetes. Develop strategies to encourage scale up and sustainability of the intervention into the standard package of services offered by government-run health centers in Sonora and other Mexican states. *Levels of context assessed*: All five levels	Mexico	Mixed method approach with a cluster-randomized trial to test effectiveness and qualitative methods to explore facilitators and barriers to adopt and integrate community health worker chronic disease interventions	Health Center participants & staff; local, state and federal policy makers.	5	NIH	https://www.gacd.org/research-projects/diabetes/dm17
**LD04: FRESH AIR–Free Respiratory Evaluation and Smoke-exposure reduction by primary Health cAre Integrated gRoups**	To improve health outcomes for people at risk of or suffering from lung diseases in LMICs through interventions for prevention, diagnosis and treatment. It uses implementation science methodologies to explore how existing knowledge and evidence-based interventions can be adapted to the practical challenges experienced in low-resource settings. *Levels of context assessed*: All five levels	Greece, Kyrgyzstan, Uganda, Vietnam	Mixed methods, action research approach including Rapid Assessments, interviews, focus group discussions and document analysis. Also questionnaires, health economic evaluation and effect measurements (for example spirometry).	Health care workers, community stakeholders (i.e. community health workers, religious leaders, village leaders), Local population with or without NCLDs.	3	EC	https://www.gacd.org/research-projects/lung-diseases/ld04
**LD05: EUREST-PLUS: Policy Implementation to Reduce Lung Diseases**	To monitor and evaluate the impact of the Tobacco Products Directive (TPD) within the context of WHO Framework Convention on Tobacco Control (FCTC) ratification at a European level. These articles in the TPD address issues of tobacco product ingredients, additives, reporting, packaging, labelling, illicit trade, cross border sales, and e-cigarettes. *Levels of context assessed*: Individual or family, community, local or district level and state or national level	Germany, Greece, Hungary, Poland, Romania, Spain	Mixed methods approach, including pre-post cohort study design; secondary data analysis of a repeated cross-sectional survey; qualitative and quantitative evaluation of e-cigarette products	Adult smokers from six EU Member States	3	EC	https://www.gacd.org/research-projects/lung-diseases/ld05
**LD15: SISTAQUIT^TM^ (Supporting Indigenous Smokers To Assist Quitting)—a cluster randomized trial to implement culturally competent evidence-based smoking cessation for pregnant Aboriginal and Torres Strait Islander smokers**	To determine whether a comprehensive culturally-competent multi-component intervention can increase quit rates in pregnant Indigenous smokers. *Levels of context assessed*: Individual or family, community, and healthcare setting	Australia	Mixed methods design to determine smoking cessation rates of pregnant patients, changes of health provider behavior in providing smoking cessation care, a health economic analysis, process measures to assess fidelity, dose, reach, recruitment and context, and qualitative data from interviews post-study to understand factors for scale-up	Health providers at Aboriginal Medical Services, and expectant mothers of Aboriginal or Torres Strait Islander babies, who are currently smoking tobacco during pregnancy	4	NHMRC	https://www.gacd.org/research-projects/lung-diseases/ld15

HT: Hypertension; DM: Diabetes; LD: Lung diseases; BP: Blood pressure; T2DM: Type II Diabetes Mellitus; CVD: Cardiovascular Disease; SMS: Short Message service; CHAP—P: Community Health Assessment Program–Philippines; CIHR: Canadian Institutes of Health Research; IDRC: International Development Research Centre; NCST: National Council of Science and Technology; National Institute of Medical Science and Nutrition Salvador Zubiran; EC: European Commission; FIC: Fogarty International Center; NIH: National Institute of Health; NHLBI: National Heart, Lung, and Blood Institute; MRC-UK: Medical Research Council, United Kingdom; NHMRC: National Health and Medical Research Council, Australia; NINDS: National Institute of Neurological Disorders and Stroke, United States

### An overview of the contextual levels assessed

**Fig 3:**
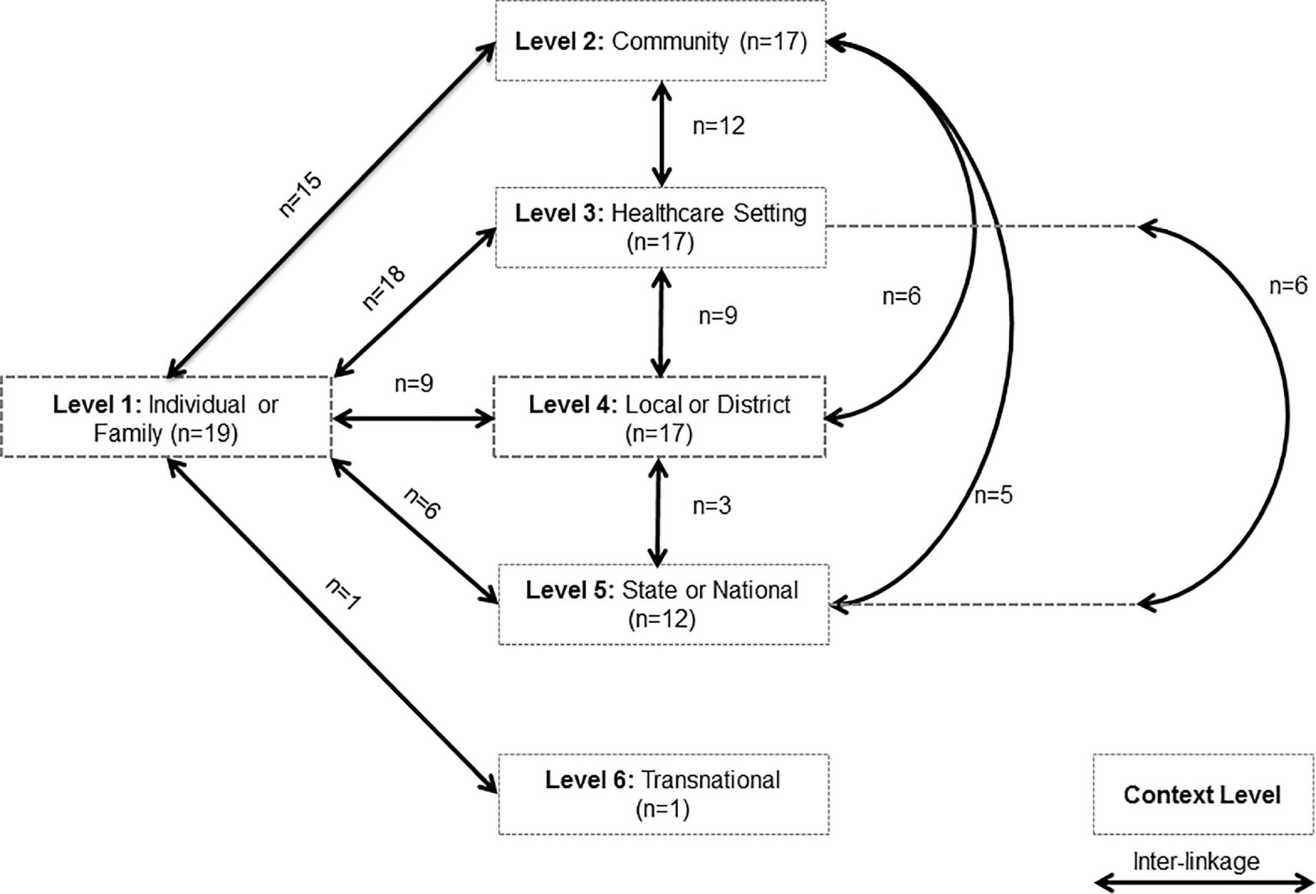
Pictorial representation of the contextual levels and inter-linkages assessed in GACD Projects (n = 20).

On average, projects assessed four of the five levels of context in the framework. Almost all of the projects (n = 19) assessed the first level of context (individual and family) and levels 2–4 (n = 17) while 12 assessed components at the state or national level (Fig 3). An additional level identified by one team, transnational i.e. comparisons of implementation between countries, was not included in the original framework. It was common (85%) to assess multiple (three or more) levels of contexts within the same project, as well as to investigate inter-linkages *between* different contextual layers. No significant differences between the research programs and number of contextual levels assessed were detected. As shown in Fig 3, the inter-linkages between the first and third (healthcare setting) level were the most frequently explored (n = 18), followed by the first and second (community level) (n = 15) and then second and third (n = 12).

Most teams used a mixed methods approach among the first three levels (76%-95%). Quantitative evaluations at baseline and end-line were more common than qualitative evaluations in the first level of context (Table 2). However, the 2^nd^-5^th^ contextual levels had more qualitative baseline and end-line evaluations. Qualitative process evaluations were commonly conducted at all (sub)levels except ‘cost of care’.

**Table 2:** Methodologies used to evaluate context at each level and sub-level.

			Baseline evaluation	Process evaluation	Endline evaluation
Level of context & sub-level	Assessed contextual level*	Mixed methods n(%)**	*Quantitative/Qualitative (n/n)†*	*Quantitative/Qualitative (n/n)†*	*Quantitative/Qualitative (n/n)†*
**1. INDIVIDUAL & FAMILY**	**19**	**18 (95)**	**15**^‡^	**13**^‡^	**15**^‡^
*Ability to Pay*	*13*	*5 (38)*	*6/5*	1/8	*6/4*
*Social Protection*	*9*	*1 (11)*	*4/4*	¼	*4/3*
*Sources of knowledge*	*16*	*8 (50)*	*8/7*	4/10	*9/4*
*Embedded social conditions*	*12*	*4 (33)*	*8/3*	2/4	*7/2*
**2. COMMUNITY**	**17**	** 13 (76)**	**13**^‡^	**13**^‡^	**11**^‡^
*Community engagement*	*14*	*5 (36)*	*2/6*	5/7	*2/4*
*Social norms*	*12*	*1 (08)*	*3/7*	1/6	*3/4*
*Sources of support*	*12*	*3 (25)*	*3/4*	3/5	*4/5*
**3. HEALTHCARE SETTING**	**17**	**14 (82) **	**13**^‡^	**13**^‡^	**11**^‡^
*Facilities & staffing*	*15*	*7 (47)*	*5/8*	5/9	*2/5*
*Cost of care*	*14*	*5 (36)*	*6/6*	6/4	*8/6*
*Organizational culture*	*9*	*2 (22)*	*2/6*	0/5	*2/5*
**4. LOCAL OR DISTRICT LEVEL**	**17**	** 6 (35)**	**13**^‡^	**8**^‡^	**9**^‡^
*Leadership & administrative practices*	*10*	*1 (10)*	*1/5*	1/5	*1/3*
*Physical environment*	*14*	*6 (43)*	*9/5*	3/6	*5/3*
***5*. *STATE OR NATIONAL LEVEL***	***12***	*** 5 (42)***	***7***^‡^	***7***^‡^	***7***^‡^
*Socio-political climate*	*6*	*0 (0)*	*1/4*	0/3	*1/3*
*National health & welfare policies*	*10*	*5 (50)*	*1/5*	3/6	*1/5*

*Includes exploration and evaluation

** % = number of mixed methods projects/total number assessed

†projects are not mutually exclusive

‡ numbers are not split by quantitative or qualitative methods

### What methodological approaches were used to assess context?

**Fig 4:**
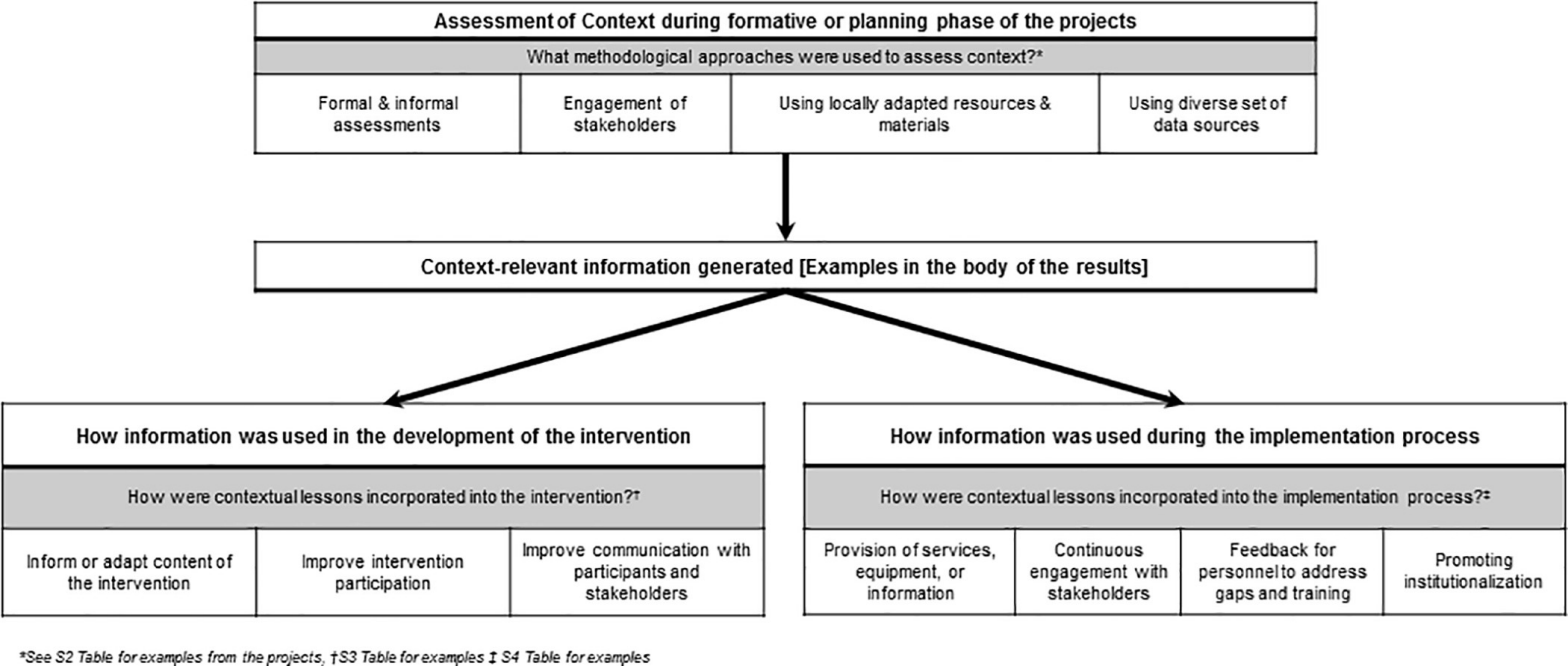
Themes identified to describe methodology or approach used to assess context and how contextual lessons are incorporated into the intervention or the implementation process.

Overall, most teams reported language translations (80%) and cultural adaptations (85%) for the tools and materials used in their intervention. In addition, four main themes representing methodological approaches (Fig 4 with examples from the project teams in S2 Table) were identified to assess context across the different levels. Specific research methods under each of the four themes (i.e. formal and informal assessments, engagement of stakeholders, using locally adapted resources and materials and using diverse set of data sources) are provided below. Approaches and frameworks from the research projects provide examples of how methods are combined and applied to assess contextual factors from varying perspectives. The contextual (sub)level where the theme was assessed is denoted in brackets directly after the theme.

#### Formal and informal assessments [all the (sub)levels of context]

From the formative to the implementation phases of the project, teams undertook various kinds of qualitative and quantitative assessments. There were four main types of assessments: situational analysis, pre/post evaluations, process evaluations, and costing. The situational analysis generally included activities ranging from informal exploration of local settings to structured assessment of needs and processes along with resource allocation and mapping. The costing assessments for example explored out-of-pocket expenditure, the ability to pay for services, and more complex costing analysis using the Socio-Technical Allocation of Resources (STAR) approach, incremental cost analysis, and comparative cost-effectiveness (e.g. facility-based versus usual care intervention). Team LD04 assessed the social protection component (individual and family level) through focus group discussions (FGDs), interviews, surveys and a stakeholder meeting. Specifically, the team explored risk factors experienced by vulnerable groups, cultural and language barriers encountered by migrant populations, hierarchical differences between patients, providers and stakeholders and receipt of social benefits such as health insurance and ration cards.

#### Engagement of stakeholders [all levels]

Arrays of participatory approaches were used to engage with various key groups related to the project ranging from classical bottom-up participatory action research to more formal stakeholder workshops. Within these participatory methods, engagement was operationalized through stakeholder meetings, consultations, or advisory panels. The DM07 team assessed community engagement by conducting stakeholder workshops and discussions during the formative phase that later guided the development of the intervention. A qualitative description of context through a topic guide facilitated a situational analysis that was based on the theoretical framework for the study. A consultative approach enabled all relevant stakeholders to be included and the knowledge gained fed into the intervention planning process.

#### Using locally adapted resources and materials [all levels]

Investigators expressed a need to create or adapt locally relevant material or resources for their specific context. This theme also included capacity building for local personnel, process for piloting or implementing the intervention, and processes for incorporating adaptations into the intervention design. The LD15 team explored the embedded social conditions sub-level by using augmented reality video and print media to be responsive to the low literacy and lower levels of education of their study population. They used a variety of role models and skin colors to be more representative of the Indigenous populations.

#### Using diverse set of data sources [first, third and fifth level]

Projects utilized diverse data sources including interviews from knowledgeable sources, administrative databases, written accounts of influential events or temporal trends that occurred throughout the project to help assess context in their setting. Approximately half of the project teams (n = 11) recorded temporal trends that could potentially influence the intervention or implementation of the study. The DM17 project assessed the socio-political climate sub-level by documenting changes in government personnel and key policies and initiatives. For example, the government leadership changed in the state where the project was implemented which resulted in several new personnel in the state health department. Further, the national government declared a state of emergency due to the emerging diabetes epidemic thus creating opportunities for related policies going forward. These events were documented to help the team understand the political environment, potential ramifications to the project and interpretation of study’s findings.

### How were contextual lessons incorporated into the intervention?

As shown in Fig 4, information generated during the assessment of the context was then incorporated into the intervention. Three main themes were identified to summarize the approaches used to incorporate the lessons: inform or adapt content of the intervention; improve intervention participation; and improve communication with participants and stakeholders (see S3 Table for specific examples).

#### Inform or adapt content of the intervention [all levels and most of the sub-components]

One of the main ways investigators incorporated contextual components into their projects was by informing or adapting their original intervention design. For example, the DM17 team adapted the physical environment context by modifying related intervention activities and designed the intervention based on the reality of access to food in the community. Their community health worker intervention to reduce cardiovascular disease risk factors initially included a recipe component where the listed ingredients could not be easily obtained in the community. The recipe was then substituted with a more appropriate one.

### Improve intervention participation [all levels except the district or state]

Targeting and maximizing participation with the intervention was one strategy project teams used to incorporate context. The approaches to improve participation ranged from promoting an all-inclusive class-free environment that encouraged access to the intervention to placing interventions in the community for easier access. For example, the DM08 team implemented one of their intervention strategies to promote healthy lifestyles in public schools in order to take advantage of freely-accessible facilities, existing infrastructure and personnel in the community.

#### Improve communication with participants and stakeholders [only sources of knowledge sub-level in first level]

Some teams sought to improve communication between the participant and other major stakeholders such as healthcare providers. The HT15 team used brief messages delivered by short message service (SMS) to provide study participants with post-clinic visit support. The team aimed to support their participants’ adoption of healthy life styles by reinforcing the new knowledge gained from the intervention.

### How were contextual lessons incorporated into the implementation process?

Fig 4 highlights the four main themes (provision of services, equipment, or information, continuous engagement with stakeholders, feedback for personnel to address gaps and training and promoting institutionalization) identified to summarize the approaches used to incorporate context into the implementation process (see S4 Table for examples).

#### Provision of services, equipment or information [all levels except local or district level]

During implementation many teams found that services, equipment, medicines or complimentary information were required to demonstrate the intervention’s success while acknowledging this was not a sustainable solution for the future. For example, the LD15 team addressed cost of care and access by providing free oral nicotine replacement therapy as this therapy was not subsidized. The HT05 team found that none of the intervention clinics had appropriately functioning equipment to measure one of their outcome variables (blood pressure). Without the equipment, there was little chance the intervention would prove to be effective. Thus, the team chose to replace the equipment in all the participating clinics. The DM04 team found through formative qualitative work that there was a lack of knowledge around eligibility for income-based programs related to diabetes management. They tailored their intervention that was structured on referral to a health centre so that those referred would be informed about access to indigent insurance programs.

#### Continuous engagement with stakeholders [all levels except at the state or national]

Project teams continually engaged with stakeholders throughout the implementation process. For example, within the leadership and administrative practices sublevel, the HT06 team incorporated context by presenting their proposed intervention to a group of government and non-government stakeholders to determine whether the proposed intervention could be integrated into the health system. This consultation was considered to be essential for eventual up-scaling. The stakeholders suggested some minor changes including training for a range of healthcare professionals in monitoring blood pressure, as well as engaging with local government at each site.

#### Feedback for personnel to address gaps and training [first three levels]

Team members reported providing feedback to personnel in order to address gaps and training needs that they found during the implementation process. The HT12 team identified hypertension knowledge gaps during a pre-assessment test and patient feedback. These gaps were subsequently incorporated into the nurse training.

#### Promoting institutionalization of the intervention [all levels]

Several teams reported being in the early stages of promoting the institutionalization of their intervention into existing systems and infrastructures. The HT15 team addressed organizational culture by re-vamping clinical information systems to generate a hospital registry. This registry had the added benefit of enabling data to be summarized on a regular basis. A task force comprising policymakers and hospital administrators were also involved in the design of the intervention building on lessons learnt from formative work involving patients and other stakeholders.

## Discussion

This paper illustrates the practical and methodological approaches used to explore, evaluate, and incorporate context into the intervention or implementation phase of implementation research projects focused on improving health in people with or at risk of chronic disease. To the best of our knowledge, this is the first exploration of how context is assessed within collaborative implementation research projects. Understanding and assessing context is particularly important when implementing health interventions in ‘real life conditions’ [22]. The results demonstrate that teams saw context (as outlined in Fig 2) as an integral part of implementation research.

### Context and implementation research

We found teams assessed four out of five contextual levels in their projects. This was anticipated as implementation research is undertaken to understand and work within real world contexts. This is in contrast to other more controlled studies in which post hoc ‘adjustments’ are made to explain what were thought to be the confounding effects of context (e.g. socioeconomic characteristics of a community). While the Medical Research Council (MRC) provides some guidelines on taking context into consideration [23], when considering the taxonomy of study designs, from efficacy trials (randomized control trials (RCTs) or cluster RCTs), effectiveness trials (pragmatic RCT) to adaptive trials (‘real world’ implementation trials), it is an intuitive assumption that the prominence of context increases the more the study design simulates ‘real-life’ settings. Thus, it is imperative to incorporate context into all phases of implementation research projects starting with the planning phase.

The GACD funding model itself also likely encourages a focus on context as the call text includes explicit emphasis on implementation and evidence-based intervention research in LMICs or vulnerable populations in HICs. It also highlights the need to understand the socio-economic, cultural, geopolitical and policy contexts and how findings might be adapted and applied in different settings [24]. The call text explicitly highlights that at the very least, there is a need for “adaptation and equitable scale-up”.

The nature and type of intervention (e.g. the target population being community, individual, healthcare workers, high-risk vs. general population etc.) would have likely influenced the levels at which context was considered and the methods used. Stakeholders are essential for eventual implementation, and so identifying effective ways to engage with them is a critical element of the contextualization process. The mode of engagement is often determined by the composition of stakeholder groups and the resulting power dynamics as described by Edmunds and Wollenberg [25] and demonstrated by some of the projects included in this study (e.g. the DM07 team shifted to a consultative approach when they realized that the stakeholders were unable to commit the time required for the initial planned participatory approach). It is well-documented that power structures within stakeholder constellations drive the type, extent and direction of change in participatory research and it is important to keep this in mind when designing interventions or implementation [26].

### Methodological considerations

The GACD network enabled access to implementation research projects representing all regions of the world and 14 different funding agencies worldwide. The fact that the scope of the study includes only projects funded through the GACD that are focused on chronic diseases is a potential limitation. However, as the first effort to document methods to assess and respond to context, the commonality of chronic disease prevention across projects is appropriate. Further, the joint funding calls ensured a clear and common definition of implementation research and the scope of research funded through each phase. The common call text developed by the funding agencies also offered a standardized consideration of context across studies funded through the GACD. Projects entered this study at different stages of their research ranging from intervention to evaluation by virtue of being funded through three different funding cycles. We minimized the differences by excluding projects that had not entered at least the intervention stage, but this may account for fewer teams describing their evaluation components.

The researchers who performed this analysis are also grant-holders which may influence the interpretation of the data. However, this bias was minimized through the reflexivity process and the feedback sought by other GACD members from projects that are not included in the study. Moreover, the Contexts and Concepts working group responsible for this work was able to leverage the expertise within the GACD Research Network and access researchers experienced in mixed methods.

GACD projects, in general, and projects represented in this study, in particular, are led by multi-disciplinary teams, a pre-requisite for implementation research. Study investigators reported a range of study designs and methods including significant use of qualitative and mixed methods, which could be explained by the fact that many of them had a formative phase, also a feature of implementation research projects. However, project management approaches were not reported, particularly methods used in quality improvement sciences or "user-centered" or agile designs [27]. Considering the scope of the survey used to collect data, it is more likely that teams did not report the project management approaches they used as the focus was on development of the intervention and implementation process. These methods are quite different compared to traditional trial-based designs and could be valuable for developing, testing, implementing and scaling-up of interventions and should be explored in further research. The scope and content of the survey also limited the data available for assessing inter-linkages; a more in-depth analysis would have shed light on a relatively unexplored aspect of context. Though structural contextual influences (e.g. racism, class, and stigma) are embedded in the socio-economic layers of context, this has not been explicitly reported by the teams included in this study. Similarly, there is a need to examine context through analytical strategies i.e. context evaluations, how it is incorporated and the effect on outcomes in a systematic manner. Finally, we should clearly acknowledge the ‘context’ of this study in its own right, i.e. diverse investigators, projects and settings with the common background of implementation science and chronic diseases.

## Conclusions

In light of the increasing awareness of the complexity of implementation research, incorporating contextual analyzes through the different stages of a project is critical to ensuring a good ‘fit’ of the intervention to the setting and the target population thereby improving the outcomes being tested. There are different approaches to assess and incorporate context as demonstrated by this study and further research is required to systematically evaluate contextual approaches in terms of how they contribute to effectiveness or implementation outcomes.

## Supporting information

S1 Table Definitions for the components of the Socio-ecological Levels of Context framework. (DOCX)

S2 Table Themes identified to describe methodology or approach used to assess context at each context level. FGDs: Focus Group Discussions; NGO: Non-governmental organizations; OOP: Out-of-pocket; STAR: Socio-Technical Allocation of Resources; EPOCH: Environmental Profile of a Community's Health; HAP: Household Air pollution; NCDs: Non-communicable diseases; PA: Physical Activity; TASSH: Task-shifting strategy for hypertension ** This table is populated with data from the open-ended questions in the semi-structured interviews to illustrate how teams reported assessing context based on the different themes identified.
(DOCX)

S3 Table Themes identified to describe how contextual lessons are incorporated into the intervention. SE: social economic; SMS: Short Message Service; T2DM:Type II Diabetes Mellitus; TASSH: Task-shifting strategy for hypertension.
(DOCX)

S4 Table Themes identified to describe how contextual lessons are incorporated into the implementation process. *This table is populated with data from the open-ended questions in the semi-structured interviews to illustrate how teams reported addressing context based on the different themes identified. FGDs: Focus Group Discussions; TASSH: Task-shifting strategy for hypertension; T2DM: Type II Diabetes Mellitus; RCT: Randomized Controlled Trial.
(DOCX)

S1 Dataset (quantitative and qualitative) and codebook. (XLSX)

## Abbreviations

ASM: Annual Scientific Meeting
EBIs: Evidence-based interventions
FGDs: Focus group discussions
GACD: Global Alliance for Chronic Diseases
HICs: High income countries
MRC: Medical Research Council
LMICs: Low and middle income countries
PARiHS: Promoting Action on Research Implementation in Health Services
RCTs: Randomized control trials
SMS: Short message service
STAR: Socio-Technical Allocation of Resources
