# Supplementary material for: The role of context in implementation research for non-communicable diseases: Answering the ‘how-to’ dilemma
Source: PLoS One. 2019 Apr 8;14(4):e0214454. doi: 10.1371/journal.pone.0214454 (PMC6453477; doi:10.1371/journal.pone.0214454)
Supplement: S1 Table — (DOCX) [file pone.0214454.s001.docx]

**S1 Table: Definitions for the components of the Socio-ecological Levels of Context** **framework.**

| **Level of Context & Sub-level** | **Definitions** |
| --- | --- |
| **INDIVIDUAL OR FAMILY** | |
| ***Ability to Pay*** | Describes the capacity to generate economic resources - through income, savings, borrowing or loans - to pay for health care services without catastrophic expenditure of resources required for basic necessities (e.g. sale of home).[26] |
| ***Social Protection*** | At the individual level, this refers to whether the individual or family is eligible for or availing any of the following:  1) “public and private initiatives that provide income or consumption transfers to the poor, protect the vulnerable against livelihood risks and enhance the social status and rights of the marginalized; with the overall objective of reducing the economic and social vulnerability of poor, vulnerable and marginalized groups”;[27]  2) Includes social policy definitions in high-income countries where social protection is usually provided by the state. It is theoretically conceived as part of the ‘state-citizen’ contract, in which states and citizens have rights and responsibilities to each other;[27] and  3) Includes private/public/employer provided health insurance or other health benefits that cover part or whole healthcare costs incurred by the individual in both HICs and LMICs. |
| ***Sources of knowledge*** | Refers to all access points used by health consumers (patients and their care givers) to better understand their health condition or support their health-related decisions. This could range from evidence-based medical opinions of experts and specialists, advice of friends and family, brochures and pamphlets from local health centers, or freely available or unauthenticated internet-based information. |
| ***Embedded social conditions*** | The situation an individual has in society because of his/her income, occupation or level of education. This includes the consequences of these social conditions such as class, stigma, discrimination, gender or power relations. |
| **COMMUNITY** | |
| ***Community engagement*** | 'Community engagement' is a planned process with the specific purpose of working with identified groups of people, whether they are connected by geographic location, special interest, or affiliation or identity to address issues affecting their collective health and well-being. |
| ***Social norms*** | Social norms are the customary rules that govern behavior in groups and societies.[28] |
| ***Sources of support*** (and degree of social support) | Social support is a protective factor for emotional and physical well-being. Sources of social support range from close and stable relations, including friends, to more distant and unstable relations and even unrelated but influential members such as a religious leader or lay health workers. |
| **HEALTHCARE SETTING** | |
| ***Facilities & staffing*** *(individual healthcare facility/setting such as primary care setting)* | Infrastructure, staffing and facilities available in healthcare for the condition under study, e.g. diabetes, hypertension, etc. |
| ***Cost of care*** *(system perspective)* | Resources utilized, or cost incurred by the healthcare system or state to provide healthcare services to its citizens. For example, systemic / incremental costs can be estimated for new interventions or policies. |
| ***Organizational culture*** *(includes organization of services and related practices including unauthorized but prevalent practices such as informal payments)* | Organizational culture is a system of shared assumptions, values, and beliefs, which governs how people dress, act and perform their jobs in organizations.[29] |
| **LOCAL OR DISTRICT LEVEL** | |
| ***Leadership & administrative practices*** | How healthcare is organized/administered/practiced at the sub-national levels, e.g. identifying the healthcare remuneration system or staff recruitment policy in the district/state in which a specific health facility belongs. |
| ***Physical environment*** | Physical conditions of the environment in which people are born, live, learn, play, work, and age that are favorable or unfavorable to an active and healthy lifestyle. |
| **STATE OR NATIONAL LEVEL** | |
| ***Socio-political climate*** | It is the aggregate, current mood and opinions of a populace on issues currently affecting that population and is influenced by both social attitudes towards and by political policies or viewpoints about the issue in question. For example, national policies governing migration create a socio-political climate in Europe that has an impact on research involving immigrant populations |
| ***National health & welfare policies*** | Refers to health and social services provided by a government for its citizens. Health policies generally include health promotion activities, primary care, and maternal and child health services. It could also include other preventive, curative or palliative services as decided by the state. Social welfare usually includes additional healthcare benefits for disadvantaged or vulnerable populations, specific subsidies or assistance for needy families, food benefits or housing assistance. Other programs, such as workers compensation, unemployment insurance and Social Security benefits are also included under social welfare policies and is variable across settings. |

**OVERARCHING THEME (applicable to all levels)**

**Temporal trends:** This is originally a concept in statistics and epidemiology, and refers to background trends which occur normally in any setting that are not initiated or governed by the intervention or implementation activities but by virtue of its influence can affect the outcome of the intervention or implementation study. For example, a sudden increase in inflation rates leading to an increase in price of fruits and vegetables or a news report about pesticide contamination of local produce. Neither of these examples are a consequence of the intervention or implementation activities but will definitely affect the outcome, if the intervention has a dietary / lifestyle component
