# Supplementary material for: The role of context in implementation research for non-communicable diseases: Answering the ‘how-to’ dilemma
Source: PLoS One. 2019 Apr 8;14(4):e0214454. doi: 10.1371/journal.pone.0214454 (PMC6453477; doi:10.1371/journal.pone.0214454)
Supplement: S2 Table — FGDs: Focus Group Discussions; NGO: Non-governmental organizations; OOP: Out-of-pocket; STAR: Socio-Technical Allocation of Resources; EPOCH: Environmental Profile of a Community's Health; HAP: Household Air pollution; NCDs: Non-communicable diseases; PA: Physical Activity; TASSH: Task-shifting strategy for hypertension ** This table is populated with data from the open-ended questions in the semi-structured interviews to illustrate how teams reported assessing context based on the different themes identified. (DOCX) [file pone.0214454.s002.docx]

**S2 Table: Themes identified to describe methodology or approach used to assess context at each context level.**

| **Context & Sub-level** | **Formal and informal assessments** | **Engagement of stakeholders** | **Using locally adapted resources and materials** | **Using diverse set of data**  **sources** |
| --- | --- | --- | --- | --- |
| **INDIVIDUAL & FAMILY (n=19)** | | | | |
| ***Ability to Pay (n=13)*** | -Estimate OOP and survey questions on how individual/family paid for healthcare costs & whether or not cost was a barrier (HT06)  -Identify contextual factors, facilitators and barriers related to sources/modes of health financing (group medical visits and microfinance groups) (DM15) |  |  |  |
| ***Social Protection (n=9)*** | Exploration to assess the situation through FGDs, interviews, surveys and stakeholder meetings (e.g. risk factors experienced by Roma; cultural and language barriers encountered by migrant populations; hierarchical differences between patients, providers and stakeholders; receipt of social benefits such as health insurance / ration cards) (LD04) |  |  |  |
| ***Sources of knowledge (n=16)*** | -Monthly process evaluation reports (DM13) -Pre-test knowledge gap on hypertension among nurses (HT12) |  | -Locally adapted awareness raising resources/materials (LD04) -State of the art searchable knowledge base for policy makers, researchers & other stakeholders (LD04) | -Collect information on messages received from healthcare providers (LD15) -Collect information on sources of knowledge (qual. work / formative phase) (DM07) |
| ***Embedded social conditions (n=12)*** | -Address knowledge & stigma level (DM10) -Collect information on education level and income (HT06) | Community advisory group discussion (DM13) | Embedding augmented reality in print media for low literacy population using models with variety of skin colors (to identify with diversity in the Indigenous population (LD15) |  |
| **COMMUNITY (n=17)** | | | | |
| ***Community engagement (n=14)*** | -Qualitative description of context using topic guide to facilitate situational analysis (DM07)  - Realist Evaluation methodology starting by developing a theory of change and refining this through qualitative exploration of context with participants (FDGs, individual interviews and participant observations) using a topic guide to focus on different levels of context (DM12) | -Collective and negotiated approach (LD15) -Consultation with key organizations and feedback on resources via expert panel [Stakeholder and Consumer Aboriginal Advisory Panel] (LD15)  - Participatory group interventions through community advisory committee and community information and consent process with community leaders (DM13) - Shifted from participatory to consultative approach when team realized the stakeholders’ expectation was to give input on the strategies (i.e. discard, accept or modify a strategy based on their understanding of its feasibility in their setting) but not design them. (DM07) -Periodic task force committee meeting with participant, study diet team, family members and relevant community members. (HT15)  -Family based approach for eating and PA habits at family and community level with the support from research team. (DM06) |  |  |
| ***Social norms (n=12)*** | -Acceptability of doing PA in the communities (HT06) -Norms towards tobacco (LD05) -Observing and understanding food habits of different cultures (DM06) |  |  |  |
| ***Sources of support (n=12)*** | -Qualitative research on social support (LD15) -Importance of key figures in family (LD15) -Assess social support from friends / family and health workers. Ask health workers about support to carry out their activities (HT06) |  | -Pilot the structure and content of the peer group sessions (DM07) |  |
| **HEALTHCARE SETTING (n= 17)** | | | | |
| ***Facilities & staffing (n=15)*** | -Economic evaluation to assess resources in terms of the local setting (HT07) -Provider interview; FGDs, facilities and services audits and facility mapping (DM13) -Improved understanding of current care process pathway for diabetes in terms of use of standardized guidelines, personnel, etc. (DM07) -Assessed available services, facilities, equipment and supplies using facility checklist evaluation (DM07) -Meeting with staff to understand the functioning of the health centers (DM10) -Determine knowledge and facilities available for hypertension (HT06) -Study clinic process (HT05) -Pre-selection and capacity building: Specify inclusion – e.g. facilities with community health nurses (HT12) |  |  |  |
| ***Cost of care (n=14)*** | -Cost evaluation using STAR approach (LD04) -Incremental cost analysis (DM07) -Comparative cost-effectiveness, e.g. facility-based vs usual care intervention (DM06) -Determining direct cost, start-up costs + maintenance costs (HT15) |  |  | -Collecting information from administrative databases (HT15) - Predicting future costs by modelling → e.g. use number of future vascular events (HT15) |
| ***Organizational culture (n=9)*** | -Semi-structured surveys and FGDs with staff on support from medical director and alienation of everyday duties due to additional project tasks; and with directors on type of support they provide (HT12) -To assess support for nurses in TASHH duties or alienation resulting from TASHH duties (HT12) | -Stakeholder meetings with all health centers pre/post intervention implementation (DM17)  Stakeholder meeting with a state government health officer, NGO, and representative from the Indian Council of Medical Research to tailor intervention suitable for scale up (HT06) |  |  |
| **LOCAL OR DISTRICT LEVEL (n=17)** | | | | |
| ***Leadership & administrative practices (n=10)*** |  | -Need for administrative back-up to establish new care pathways which are not regarded as administrative practice change, but in reality, cannot be done without administrative support (DM07) -Stakeholder meetings including state health departments and jurisdictional leadership (DM17) | - The initial message set was developed using stakeholders (clinicians, managers and other administrative staff) the health department and official disease management guidelines, we then tested the content and tone using focus groups of patient-participants, the message set was refined through this process and then retested and translated. (DM12) | - Trial diary of individual researchers at each study site to track of relevant local policies that affect the project implementation e.g. changes in administrative responsibilities for primary care at county level (DM07) |
| ***Physical environment (n=14)*** | -Identify critical factors for successful implementation (LD04) -Village mapping of healthy and unhealthy spaces (DM13) -Food environment evaluation using EPOCH tool (DM07) -Realist evaluation methodology (DM12) - Assessment of how the environment limits our ability to exercise or access health care (HT06) | -Stakeholder consultation as part of the process evaluation was used to assess the potential to influence the food supply through the intervention (HT10)  - Semi-structured interviews were used to understand the impact of the cyclone on the intervention (HT10) |  |  |
| **STATE OR NATIONAL LEVEL (n=12)** | | | | |
| ***Socio-political climate (n=6)*** | -Differential implementation of EU legislation in the national environment (LD05) |  |  | - Change in leadership (new governor) and declaration of state of emergency (DM17) - Written record of key temporal changes that would affect intervention or project implementation, e.g. changes in administrative practices and jurisdictions of health regions (DM17) |
| ***National health & welfare policies (n=10)*** | -Changing role definitions → e.g. involving community health workers for NCDs, feasibility and adaptability issues (HT07)  - Policy mapping was used as the outset of the project to understand where we could integrate the intervention into existing policies(HT10) | - Group and individual consultations with national experts and practitioners on diabetes diagnostics and prevention and treatment strategies, including nutrition advice and service guidelines (DM13)  - State policy maker consultations (DM04)  - National NGO (e.g. national endocrine society) consultations (DM04) | -International consortium with key policy expertise (LD04) |  |

* FGDs: Focus Group Discussions; NGO: Non-governmental organizations; OOP: Out-of-pocket; STAR: Socio-Technical Allocation of Resources; EPOCH: Environmental Profile of a Community's Health; HAP: Household Air pollution; NCDs: Non-communicable diseases; PA: Physical Activity; TASSH: Task-shifting strategy for hypertension ** This table is populated with data from the open-ended questions in the semi-structured interviews to illustrate how teams reported assessing context based on the different themes identified.
