# Supplementary material for: The role of context in implementation research for non-communicable diseases: Answering the ‘how-to’ dilemma
Source: PLoS One. 2019 Apr 8;14(4):e0214454. doi: 10.1371/journal.pone.0214454 (PMC6453477; doi:10.1371/journal.pone.0214454)
Supplement: S3 Table — SE: social economic; SMS: Short Message Service; T2DM:Type II Diabetes Mellitus; TASSH: Task-shifting strategy for hypertension. (DOCX) [file pone.0214454.s003.docx]

**Supplementary Material**

| **S3 Table: Themes identified to describe how contextual lessons are incorporated into the intervention.** | | | |
| --- | --- | --- | --- |
| Level of Context and Sub-level | **Inform or adapt content of the intervention** | **Improve intervention participation** | **Improve communication with participants and stakeholders** |
| **INDIVIDUAL & FAMILY (n=19)** | | | |
| **Ability to Pay (n=13)** |  | - Intervention adjusted to allow participation across all SE strata (LD04) and cost-effective, while being locally relevant (HT15)  - Intervention implemented in public primary schools to use freely-accessible facilities/infrastructure and personnel in the community. (DM08) |  |
| **Social Protection (n=9)** |  |  |  |
| **Sources of knowledge (n=16)** | - Inform intervention elements (content and delivery) based on formative phase (DM07)  - Generate an explanatory model based on patient perception e.g. to help the team design messages about diabetes detection and management (DM04)  - Educational video addresses gaps e.g. importance of prompt referral (HT15) |  | - [As part of the intervention] Improve communication and consistency of message from provider to patient (LD15)  - Study participants receive post-clinic visit brief messages to promote the adoption of healthy life style (delivered using SMS text message) (HT15) |
| **Embedded social conditions (n=12)** | - Through discussions with community advisory groups and the participatory learning (DM13)  - Simplified educational materials, mainly pictorial (HT06) | - Recruit participants from all social strata (HT15)  - Create an all-inclusive social class-free environment that encourages unhindered access to the benefits of the intervention (HT15) |  |
| **COMMUNITY (n=17)** | | | |
| **Community engagement (n=14)** | - Test [intervention] resources in separate FGDs with healthcare workers and community leaders (LD04)  - Piloting of message with community advisory groups (m-health) (DM13)  - Used assess-develop-test-refine-test with the lens of a health condition (e.g. T2DM), health care (organization and access including ability to pay), and social circumstances (including transport, food security, employment) (DM12) | Group-based education program located in the community enabling better access (HT06) |  |
| **Social norms (n=12)** | Formative research learnings fed back into the intervention within the context of existing social norms (e.g. observing and understanding different countries food habits then gives us the knowledge to understand how to tailor intervention in different settings) (DM06) | Involve families of pregnant women in the intervention & assessing smoke-free homes (LD15) |  |
| **Sources of support (n=12)** | - Incorporating learnings into messages and content of mHealth and group interventions, e.g. by giving examples/models of opportunities for community support for the prevention and control of disease and how to access it (DM13)  - Modifying intervention [role of family] and materials [for social mobilization] (DM07) | Involve family and caregivers [of stroke patients] to ensure better attendance of clinic visits and uptake of the intervention [to prevent recurrent vascular events] (HT15) |  |
| **HEALTHCARE SETTING (n=17)** | | | |
| **Facilities & staffing (n=15)** | Tailoring of intervention (DM04) based on type of facility (rural or urban health centre) and types of staff available (midwives, physicians community health workers) | Use of Accredited Social Health Activist (ASHA – community selected health workers) to implement intervention (HT06) |  |
| **Cost of care (n=14)** | Tailor intervention → e.g. choose the type and process for blood pressure measurement (DM04) |  |  |
| **Organizational culture (n=9)** |  |  |  |
| **LOCAL or DISTRICT (n=17)** | | | |
| **Leadership and administrative practices (n=10)** | Intervention tailored accordingly based on feedback from major’s office so that very rural remote locations were not included (DM04) |  |  |
| **Physical environment (n=14)** | - Modification of intervention based on reality of access to food and exercise opportunities in the community (DM17)  - Intervention tailored accordingly based on roving across different local settings and using whatever buildings or inside/outside space available to set up the intervention. (DM04)  - Enable participants to tackle the food environment (DM07)  - Revision of dietary and sections of recommendations (patient report card PRC) and the scoring of progress (HT15)  - Existing information [awareness, personal determinants (awareness, beliefs) and resources] will help determine appropriate interventions (LD04) |  |  |
| **STATE or NATIONAL (n=12)** | | | |
| **Socio-political climate (n=6)** | Tailoring intervention accordingly → e.g. timing / who is involved, etc (DM04) | Used the information to decide on how to introduce the intervention to the directors of the health centers (DM17) |  |
| **National health & welfare policies (n=10)** |  |  |  |
| SE: social economic; SMS: Short Message Service; T2DM:Type II Diabetes Mellitus; TASSH: Task-shifting strategy for hypertension | | | |
