# Supplementary material for: The role of context in implementation research for non-communicable diseases: Answering the ‘how-to’ dilemma
Source: PLoS One. 2019 Apr 8;14(4):e0214454. doi: 10.1371/journal.pone.0214454 (PMC6453477; doi:10.1371/journal.pone.0214454)
Supplement: S4 Table — *This table is populated with data from the open-ended questions in the semi-structured interviews to illustrate how teams reported addressing context based on the different themes identified. FGDs: Focus Group Discussions; TASSH: Task-shifting strategy for hypertension; T2DM: Type II Diabetes Mellitus; RCT: Randomized Controlled Trial. (DOCX) [file pone.0214454.s004.docx]

**S4 Table: Themes identified to describe how contextual lessons are incorporated into the implementation process.**

| Level of Context and Sub-level | **Provision of services, equipment, or information** | **Continuous engagement with stakeholders** | **Feedback for personnel to address gaps and training** | **Promoting institutionalization** |
| --- | --- | --- | --- | --- |
| **INDIVIDUAL & FAMILY (n=19)** | | | | |
| **Ability to Pay (n=13)** | Health insurance coverage (HT12), free education sessions, information on affordable medication (HT06) |  |  |  |
| **Social Protection (n=9)** | Use multiple sources to provide relevant information to participants (eligibility for income-based programs; information on affordable medication through practitioners) (DM04) | Stakeholder participation in implementation with routine feedback & engagement with existing community-based governance structures for sustainable solutions (e.g. development of microfinance intervention) (DM15) |  |  |
| **Sources of knowledge (n=16)** | - Select appropriate personnel to provide info e.g. Community-based health care workers or other volunteers for provision of care (HT07)  - Inform routine implementation of an in-clinic education video to address gaps in stroke knowledge (HT15) | Used a dynamic participatory approach e.g. to address myths / misconceptions on stroke (HT15) | **-** Feedback to practitioners and administrative staff  - Educational video addresses gaps (importance of prompt referral) which was used to trained personnel (HT15)  - Hypertension knowledge gaps identified and incorporated into nurse training (HT12)  - Based on feedback from patients e.g. nurses encouraged to clearly explain the condition and management strategy (HT12) |  |
| **Embedded social conditions (n=12)** | Inform about alternatives e.g. procure fruits and vegetables from farms if they are not able to obtain healthy food (HT12) |  | **-** Interviews were conducted with health providers who underwent the webinar training and participated in the pilot study to inform the full RCT and improve implementation (LD15)  - Feedback to practitioners, staff and administrators of some social factors that affect follow-up (DM10) | Advocacy for institutionalization of a stroke support group (HT15) |
| **COMMUNITY (n=17)** | | | | |
| **Community engagement (n=14)** | Inform on where to find affordable medicines (HT06) | - Engage [Aboriginal] community (LD15)  - Assessing progress of the implementation throughout together with communities (DM04) |  |  |
| **Social norms (n=12)** | Provide practical description on how to do physical activities in their own homes (HT06) |  |  | Using health centers to conduct study and integrate the intervention into existing programs (DM17) |
| **Sources of support (n=12)** | - Developing materials for social mobilization by using stakeholder workshops (DM07)  - Incorporate findings and provide augmented social support from key figures [family and the community] for study participants [pregnant smokers] (LD15)  - Encourage and provide information to women on how to access social support and education (LD15)  - Community health nurses encouraged to provide support in other spaces than healthcare (HT12) | - Engagement with community / religious leaders (DM13) | Administrators and physicians were informed that many patients required individual psychological support. In the past, occasional group support was granted. Social workers were asked to provide more support for supplies (strips) for glucometers by the health provider. (DM10) |  |
| **HEALTHCARE SETTING (n=17)** | | | | |
| **Facilities & staffing (n=15)** | - Resources are adapted to local context e.g. healthcare infrastructure (LD04)  - Pilot trial findings apply to larger project [stratify or match communities by health care resource levels] (LD15)  - Provided equipment (e.g. cuffs for the electronic blood pressure machine) to the clinics in order to measure the outcome variable (blood pressure) (HT05)  - Alternative provision of services utilized due to lack of services at the healthcare facility (e.g. health workers used in the community for patient education) (HT06)  - Provide lab equipment, supplies, etc. (in case of non-functioning labs) (HT12) |  | Training of nurses to diagnose and manage hypertension (HT12) | - Service training for all staff [for smoking cessation care] (LD15)  - Integrate intervention into existing programs in the participating health centers (DM17)  - Findings to be communicated to national government bodies and decision makers (DM06) |
| **Cost of care (n=14)** | - Provide free oral nicotine replacement therapy as it is not subsidized (LD15)  - Provision of free services during trial →e.g. medication, HbA1c, nurse visits, confirmatory testing, insurance to participants (DM07)  - Provide health insurance – cost cited as a barrier to receiving health care (HT12) | Demonstrate intervention is cost-effective model to stakeholders (LD04) |  | - Integrate intervention into existing programs (DM17)  - Report finding to national bodies (DM06) |
| **Organizational culture (n=9)** |  |  | - Building and strengthening capacity of health workers (HT15)  - Engage more with sites which seemed to provide minimal support to nurses (for TASSH duties) (HT12) | Re-vamping clinical information systems by generating hospital registry → added advantage that study will then generate data regularly (HT15) |
| **LOCAL or DISTRICT (n=17)** | | | | |
| **Leadership and administrative practices (n=10)** |  | Consultative process → determine if proposed intervention would fit into the health system and how to modify (HT06) |  | Consultative process → determine if proposed intervention would fit into the health system and how to modify (HT06) |
| **Physical environment (n=14)** |  |  |  | Informed approach to industry [through legislation and voluntary targets] (HT10) |
| **STATE or NATIONAL (n=12)** | | | | |
| **Socio-political climate (n=6)** |  |  |  | Informs regulatory approach (HT10) |
| **National health & welfare policies (n=10)** | Identify gaps / shortages in service delivery in relation to ‘ideal’ services (DM13) |  |  | - Aligned intervention to relevant national and international policies and protocol (DM13)  - Basis of evidence-informed recommendations (HT15)  - Serve as a model to be implemented at a national level (HT15)  - Integrate intervention into existing policies and programs (HT10) |
| *This table is populated with data from the open-ended questions in the semi-structured interviews to illustrate how teams reported addressing context based on the different themes identified. FGDs: Focus Group Discussions; TASSH: Task-shifting strategy for hypertension; T2DM: Type II Diabetes Mellitus; RCT: Randomized Controlled Trial | | | | |
